# Comparative Efficacy and Toxicity of Different Species of* Sargassum* in Haizao Yuhu Decoction in PTU-Induced Goiter Rats

**DOI:** 10.1155/2017/3526186

**Published:** 2017-06-21

**Authors:** Linlin Xiu, Gansheng Zhong, Dianna Liu, Shaohong Chen, Haiyan Liu, Feng Chen

**Affiliations:** Beijing University of Chinese Medicine, Beijing, China

## Abstract

**Background:**

Haizao Yuhu Decoction has been widely used to treat thyroid-related diseases especially goiter with few side effects in traditional Chinese medicine (TCM), including herb pair* Sargassum* (HZ) and Glycyrrhizae Radix et Rhizoma (GC), as one of “eighteen antagonistic medicaments.” The two different species of* Sargassum*,* Sargassum fusiforme* (Sf) and* Sargassum pallidum* (Sp), are not clearly differentiated in clinical use, so that herb pair Sf-GC and Sp-GC could show different effect and toxicity.

**Methods:**

We investigated the antigoitrous effect and toxicity and clarified the potential underlying mechanism of the two different species of* Sargassum* in HYD (HYDf and HYDp) in PTU-reduced goiter rats.

**Results:**

The results demonstrated that both HYDf and HYDp could exhibit antigoitrous effect through alterations in hypothalamus-pituitary-thyroid (HPT) axis and inhibition of the TPO gene expression; there is no difference in the antigoitrous effects between the two different species of* Sargassum* application in HYD.

**Conclusion:**

This study evaluated the safety and efficacy of herb pair HZ-GC applied in HYD in goiter rats at molecular, cellular, and whole level and compared the two species of* Sargassum* further. We provide a reliable way to clarify the possible mechanism of the antagonistic medicament herb pair HZ-GC for its application.

## 1. Introduction

Goiter, an enlargement of the thyroid gland, is a common problem in clinical practice [1]. It can be associated with a number of thyroid diseases such as thyroid dysfunction (hyperthyroidism and hypothyroidism), autoimmune thyroid disease (Graves' disease and Hashimoto's thyroiditis) [2, 3], thyroiditis, and thyroid cancer [4, 5]. Regardless of varies clinical manifestation, the etiology of goiter is essentially the same: the thyroid attempting to adapt to circumstances of changes in the thyroid's ability to secrete adequate amounts of hormones [1], of which hypothyroidism is the most common in recent decade [6]. Hypothyroidism, abnormalities in thyroid hormone synthesis or thyroid dyshormonogenesis, is a common endocrine disorder [7] involved with multiple organs. The single most common cause of hypothyroidism worldwide was undoubtedly iodine deficiency. Where it is not a problem, the most common causes are chronic autoimmune thyroiditis and previous thyroid surgery or ^131^I therapy. Its clinical characteristics are complex. Internationally, conventional drugs, such as potassium iodide and levothyroxine, are recommended for patients with hypothyroidism. However, their respective curative effects remain controversial [8].

According to traditional Chinese medicine (TCM) theory, goiter is mainly due to “Qi” stagnation and “phlegm” stasis [9]. Haizao Yuhu Decoction (HYD), which is described in a famous TCM monograph Waike Zhengzong (Summary of Surgical Medicine) in Ming Dynasty of China, has been used for approximately 500 years and famous for its efficacy in treating thyroid-related diseases especially goiter based on the clinical study [10]. It is worthwhile to mention that there is a herb pair* Sargassum* (Haizao, HZ) and Glycyrrhizae Radix et Rhizoma (Gancao, GC), one of the so-called “eighteen antagonistic medicaments” in TCM documents, indicating that the two herbs should not be applied simultaneously, whereas the herb pair HZ-GC has been widely used in HYD for the treatment of thyroid dysfunction, galactophorous hyperplasia, and ovarian cyst [11]. In recent decades, some literature research, clinical study, and animal experiment have been carried out to evaluate the efficacy and safety of the combination [12, 13]. However, their respective curative effects and mechanisms remain controversial and there is limited scientific evidence to establish the safety and efficacy of these herbal products.

Additionally,* Sargassum fusiforme* (Yangqicai, Sf) and* Sargassum pallidum* (Haihaozi, Sp) are two different species of* Sargassum* officially recorded in the Chinese Pharmacopoeia but not clearly differentiated in clinical use. The pharmacological effects of them have been extensively studied [14], but there are few comparative studies between them.

What roles do these two different species of* Sargassum* play in the herb pair HZ-GC, and whether the different species are related to impact effects of HYD? Accordingly, we proposed the present study to systematically evaluate antigoitrous effect and toxicity and to clarify the underlying mechanism of the two different species of* Sargassum* in HYD in PTU-reduced goiter rats at molecular, cellular, and whole level.

## 2. Materials and Methods

### 2.1. Preparation of Haizao Yuhu Decoction

HYD is a decoction of 12 Chinese herbs as listed in Table 1.

As there are two species of* Sargassum*, Haizao Yuhu Decoction with the species of* Sargassum fusiforme* (HYDf) and Haizao Yuhu Decoction with the species of* Sargassum pallidum* (HYDp) were prepared separately. The rat equivalent doses were selected based on the clinical adult dosage provided by the Chinese Pharmacopoeia and calculated according to the ratio of body-surface area between human and rat.

All herbs used in the study were purchased from Beijing Shuangqiao Co., Ltd. (Beijing, China) and Anhui Tongling Co., Ltd. (Tongling, China) and authenticated in accordance with the standard of the Chinese Pharmacopoeia by Professor Li (School of Basic Medical Science, Beijing University of Chinese Medicine). The mixtures were soaked in distilled water in a ratio of 1/10 (g/ml) for 2 h and then subsequently boiled for a further 60 min. After saving the supernatant collected by filtering, the residue was added with a ratio of 1/8 (g/ml) distilled water and boiled again for a further 30 min. Finally, the two portions of aqueous extracts were pooled together and concentrated to a density of 1.0 g/ml crude herb by means of heating evaporation.

### 2.2. Experimental Reagents

Propylthiouracil (PTU) was purchased from Zhaohui Pharmaceutical Technology Corporation (Shanghai, China); Euthyrox was purchased from MerkKGaA (Darmstadt, Germany).

### 2.3. Preparation of Goiter Model and Grouping

50 Male and female Wistar rats (180–220 g) were purchased from Charles River Laboratories, Beijing, China (Certificate of Conformity: SCXK (Beijing) 2012-0001). The animals had free access to drinking water and regular chow with a standard commercial rat feed every day and were housed in a room under controlled conditions (temperature 22 ± 2°C, humidity 50 ± 10%, and light illumination 12 h/d) (Certificate of Conformity: SCXK (Beijing) 2011-0024). Experimental protocols were approved by the Experimental Animal Care and Ethics Committees of Beijing University of Chinese Medicine.

After 3 days of acclimatization, the rats were randomly divided into 5 groups: normal, model, Euthyrox, HYDf, and HYDp groups; all rats except those in the normal control group received intragastric administration of PTU at a dosage of 0.01 g/kg daily for 14 days to induce the goitrous model according to previously reports [15] and were given different treatments for 28 days, as shown in Table 2.

### 2.4. Sample Collection

After 28 days of treatment, diets were removed from the cages 12 h before the animals were sacrificed. Blood samples were collected and centrifuged at 3000 ×g for 12 min to obtain serum.

Thyroid glands, liver, and kidney were isolated, weighed, and stored at −80°C for pathological observation.

### 2.5. General Observations

Clinical signs and mortality were recorded twice a day (before and after treatment) throughout the study period. The body weight (BW) and rectal temperature of each rat were measured at the initiation of treatment and once a week during the study period.

### 2.6. Thyroid Function Assessment, Relative Organ Weights Assessment, and Biochemical Analysis

Absolute organ (thyroid gland, liver, and kidney) weights were measured and relative organ weights (organ-to-body weight ratios, g/100 g BW) were calculated.

The levels of serum free triiodothyronine (FT3), free thyroxine (FT4), thyroid stimulating hormone (TSH), and thyrotropin releasing hormone (TRH) were determined by radioimmunoassay using commercially available kits (purchased from Biosino. Inc., Beijing, China) according to manufacturer's instructions.

Serum alanine aminotransferase (ALT), aspartate aminotransferase (AST), alkaline phosphatase (ALP), blood urea nitrogen (BUN), creatinine (Cr), and uric acid (UA) were determined to assess liver and kidney function using commercially available kits (purchased from Biosino. Inc., Beijing, China) according to manufacturer's instructions.

### 2.7. Histopathology

After all animals had been sacrificed, thyroid glands were removed and divided into two parts; one was put in a buffer solution of 10% formalin and embedded in paraffin. Six to ten 4 *μ*m-thick sections were prepared in a noncontiguous way and dyed with hematoxylin-eosin (H-E); the stained areas were viewed using an optical microscope to assess the pathologic alterations of thyroid tissues in different groups and to evaluate the effects of the treatments.

Another one was immediately stored in liquid nitrogen at −80°C for real-time reverse transcription-polymerase chain reaction (RT-PCR) analysis.

### 2.8. RT-PCR Analysis

Thyroglobulin (Tg) mRNA and thyroid peroxidase (TPO) mRNA expression were determined using RT-PCR. Total RNA was extracted from thyroid glands using the Trizol reagent (Invitrogen, USA). RNA yields and purity were assessed by spectrophotometric analysis (Spectronic Unicam, USA). Total RNA (1 *μ*g/*μ*l) transcription was performed using an in vitro transcription Kit (PrimeScript RT reagent Kit Perfect Real Time, TaKaRa, Japan). RT-PCR reactions were performed with 20 *μ*l reactions that consisted of 10 *μ*l 2x Taq PCR MasterMix (Solalbio, Beijing, China), 0.5 *μ*l PCR forward primer (10 *μ*M), 0.5 *μ*l PCR reverse primer (10 *μ*M), 3 *μ*l cDNA, 0.5 *μ*l GAPDH forward primer, 0.5 *μ*l GAPDH reverse primer, and 5 *μ*l double-distilled water. The PCR cycling conditions comprised a denaturation step for 3 min at 94°C, followed by 34 cycles of denaturation (94°C for 30 s), annealing (65°C for TPO, 62°C for Tg and 56°C for GAPDH for 30 s), and extension (72°C for 1 min). After the last cycle, all PCR products were subjected to a final extension for 8 min at 72°C. The primer sequences were shown in Table 3.

PCR products were combined and then electrophoresed on 1.5% agarose gels containing ethidium bromide; data analysis was carried out using Alpha Ease FC software (Alpha Intech, USA). Values obtained for Tg and TPO were normalized against values obtained for GAPDH, and the results were expressed as relative integrated intensity.

### 2.9. Statistical Analysis

The data was analyzed using the SPSS 21.0 software and expressed as the mean ± standard deviation (SD). Differences between groups were examined for statistical significance using one-way analysis of variance (ANOVA; SPSS 20.0 for Windows; SPSS Inc., USA). The results were considered statistically significant when *P* < 0.05.

## 3. Results

### 3.1. Body Weight and Temperature Changes in PTU-Induced Goiter Rats

**Figure 1:**
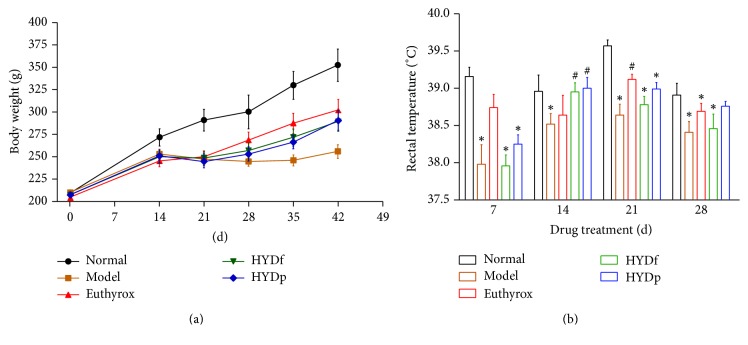
(a) Mean body weight changes in PTU-induced goiter rats. (b) Temperature changes in PTU-induced goiter rats after 28-day drug treatment. Data are presented as mean ± SD, ^*∗*^*P* < 0.05 versus normal; ^#^*P* < 0.05 versus model.

Body weight of rats was increased in a time-dependent manner (Figure 1(a)). PTU treatment for 14 days resulted in marked decreases in body weight and rectal temperature (Figure 1).

There were statistical increases of body weight in Euthyrox, HYDf, and HYDp treatment groups compared to the model group after 28 days drug treatment (*P* < 0.05). However, no difference was observed on body weight change in HYDf and HYDp.

After drug treatment for 28 days, there were obvious decreases of rectal temperature in the model, Euthyrox, and HYDf treatment groups compared to the normal group (*P* < 0.05). The rectal temperature of HYDp treatment group was statistically higher than that of HYDf treatment group (*P* < 0.05).

### 3.2. Comparison of Relative Thyroid Weights and Serum Levels of FT3, FT4, TSH, and TRH

**Figure 2:**
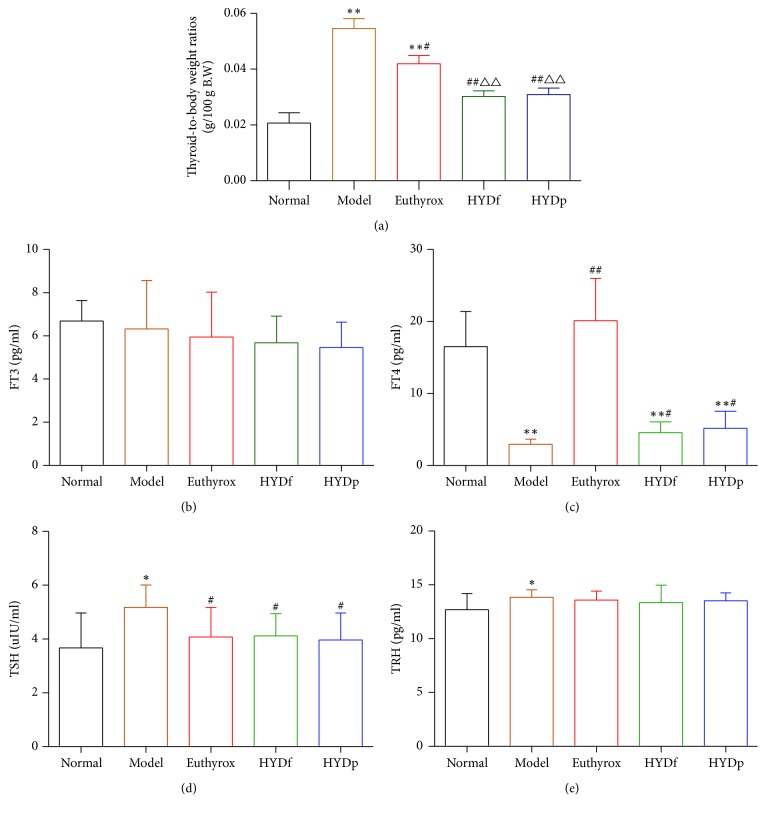
Comparison of relative thyroid weights (a), serum levels of FT3 (b), FT4 (c), TSH (d), and TRH (e). (a) Thyroid enlargement of goiter rats could be significantly suppressed by Euthyrox, HYDf, and HYDp treatment. HYDf and HYDp showed significant effect on the PTU-induced goiter rats, which were better than that of positive control Euthyrox. (b) No difference was observed on serum levels of FT3 change in different treatment groups. (c) The reduced serum levels of FT4 in goiter rats were increased in Euthyrox, HYDf, and HYDp treatment groups. (d), (e) The elevated serum levels of TSH and TRH in goiter rats were decreased by their treatment. Data are represented as the mean ± SD, ^*∗*^*P* < 0.05 and ^*∗∗*^*P* < 0.01 versus normal; ^#^*P* < 0.05 and ^##^*P* < 0.01 versus model; ^∆∆^*P* < 0.01 versus Euthyrox.

Relative thyroid weights of model group were significantly increased (*P* < 0.01), its serum FT4 levels were significantly lower than those of the normal controls (*P* < 0.01), and serum TSH levels were higher than those of the normal controls (*P* < 0.05), indicating the successful production of PTU-induced hypothyroidism goiter model (Figure 2).

Euthyrox, HYDf, and HYDp showed a significant effect on the PTU-induced goiter rats after 28-day treatment (Figure 2(a)). In addition, thyroid enlargement was suppressed significantly in HYDf and HYDp treatment groups compared to Euthyrox group (*P* < 0.01).

The reduced levels of FT4 in the serum of goiter rats could be increased by Euthyrox, HYDf, and HYDp (*P* < 0.01; *P* < 0.05, Figure 2(c)). In addition, the elevated serum levels of TSH and TRH in goiter rats were decreased by their treatments (*P* < 0.05, Figures 2(d) and 2(e)).

### 3.3. Comparison of Some Liver/Renal Function Parameters

**Figure 3:**
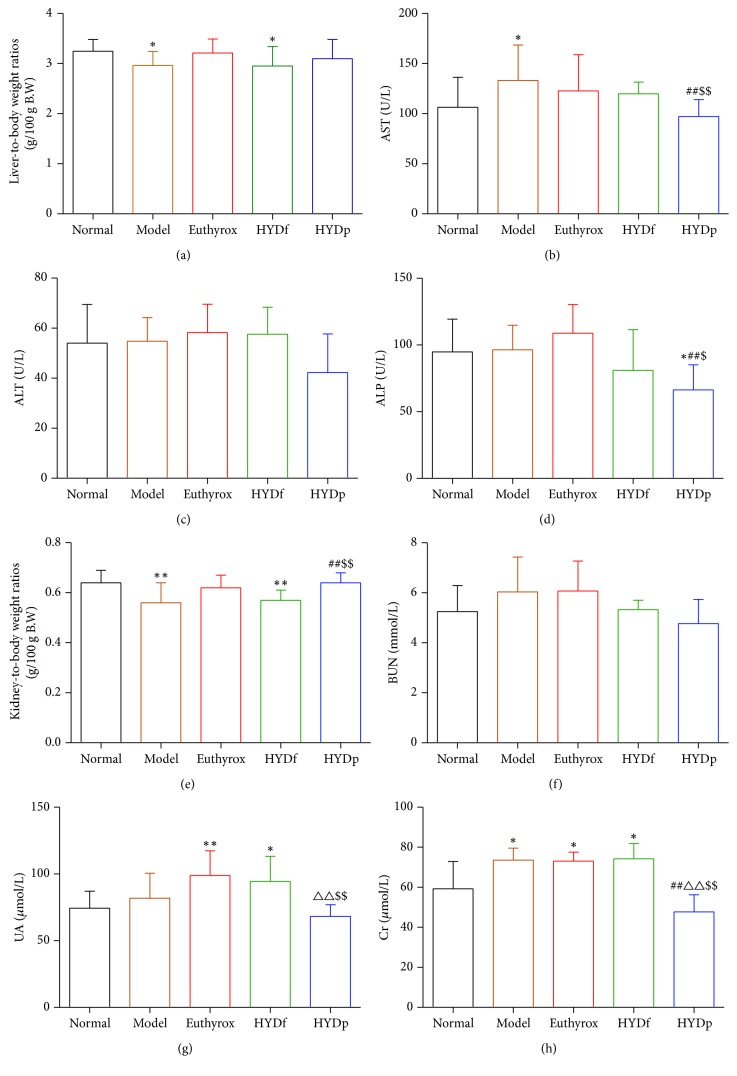
Comparison of relative liver weights (a), serum activities of AST (b), ALT (c), ALP (d), relative kidney weights (e), serum levels of BUN (f), UA (g), and Cr (h). Data are presented as mean ± SD, ^*∗*^*P* < 0.05 and ^*∗∗*^*P* < 0.01 versus normal; ^##^*P* < 0.01 versus modle; ^∆∆^*P* < 0.01 versus Euthyrox; ^$^*P* < 0.05 and ^$$^*P* < 0.01 versus HYDf.

Compared to the normal controls, relative liver/kidney weights of model group were decreased (*P* < 0.05, Figure 3), and its serum activities of AST and Cr were obvious higher (*P* < 0.05); serum UA and Cr levels of Euthyrox treatment group were higher (*P* < 0.01; *P* < 0.05); relative liver/kidney weights of the HYDf treatment group were decreased (*P* < 0.05), and its serum UA and Cr levels were higher (*P* < 0.05).

The HYDp treatment group significantly improved the relative renal weights as well as the activities of AST, ALP, and Cr compared to the goiter model group (*P* < 0.01).

Besides, compared to the HYDf treatment group, the HYDp treatment group significantly improved the relative renal weights and the serum levels of AST, ALP, UA, and Cr (*P* < 0.01).

### 3.4. Histological Observations of the Thyroid Gland in Different Groups

**Figure 4:**
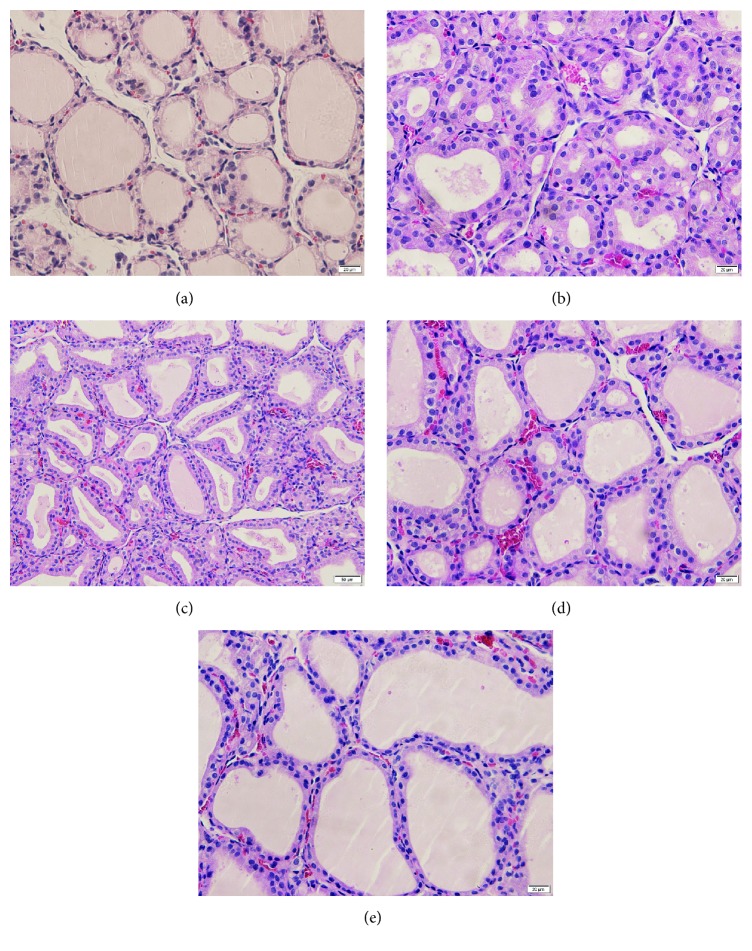
Histology of thyroid gland (H-E staining 400x). (a) Normal control. (b) Goiter model. (c) Goiter rats treated with Euthyrox. (d) Goiter rats treated with HYDf. (e) Goiter rats treated with HYDp.

Structurally, thyroid gland in PTU-induced goiter model group showed a diffuse homogeneous pattern of follicular epithelial cells hyperplasia with limited volumes of stored colloid compared to normal control group (Figure 4). Although the physiological parameters had returned to normal values, thyroid gland in the Euthyrox treatment group did not return to a morphologically euthyroid state (Figure 4(c)), which was consistent with the results of relative thyroid weights assessment. In contrast, follicular cell hyperplasia of the thyroid gland was obviously improved by HYDf and HYDp treatment (Figures 4(d) and 4(e)); no remarkable differences were found between the two groups.

### 3.5. Tg mRNA and TPO mRNA Levels of Different Groups

**Figure 5:**
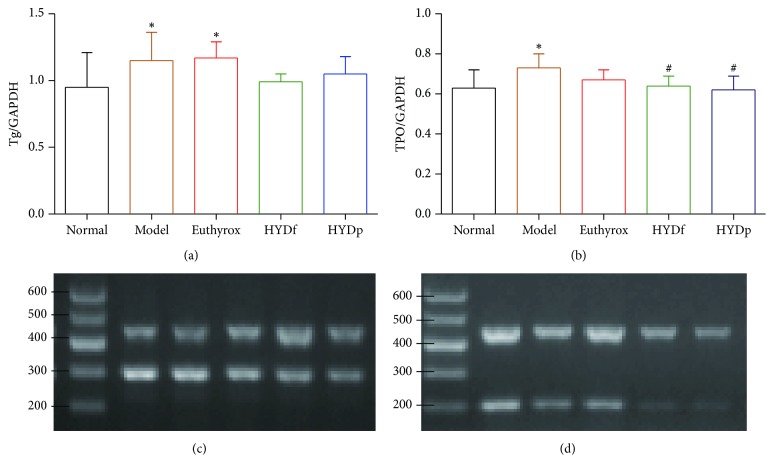
Tg mRNA (a, c) and TPO mRNA (b, d) levels of different groups. Data are presented as mean ± SD. ^*∗*^*P* < 0.05 versus normal; ^#^*P* < 0.05 versus model.

RT-PCR analysis showed that the mRNA levels of Tg and TPO expression in thyroid tissues of the goiter model group were both higher than those in normal group (*P* < 0.05). The administration of HYDf and HYDp could markedly decrease TPO expression compared to model group (*P* < 0.05, Figure 5).

## 4. Discussion

Haizao Yuhu Decoction has been widely used to treat thyroid-related diseases especially goiter with few side effects in TCM. Although the mechanisms are not clear so far, it is considered as a multistep process involving thyroid hormone synthesis, antioxidative stress, and immunomodulatory effect [14, 16]. Notably, herb pair HZ-GC, as one of “eighteen antagonistic medicaments” which means that the two herbs should not be used in the same prescriptions in order to avoid mutually, has been demonstrated regulating targets into the synthesis of thyroid hormone [16], indicating the critical roles of this herb pair in HYD acting on goiter. The two different species of* Sargassum*, Sf and Sp, were studied for their pharmacological effects and acute toxicity [12]. The results of this initial study clearly demonstrated that there were marked differences in their chemical components and acute toxicity.

Therefore, we speculated that the use of different species of* Sargassum* in formula compound HYD (HYDf and HYDp) could have different pharmacological effects, accordingly, as for herb pair HZ-GC, the use of different species of* Sargassum*, herb pair Sf-GC, and herb pair Sp-GC could show different efficacy and toxicity, which might relate to the antagonistic medicament. To investigate whether the use of different species of* Sargassum* is related to antagonistic medicament, we proposed the present study to systematically evaluate antigoitrous effect (FT3, FT4, thyroid weight, and histological changes) and toxicity (liver/renal function parameters) and to clarify the potential underlying mechanism (TSH, TRH, Tg mRNA, and TPO mRNA) of the two different species of* Sargassum* in HYD (HYDf and HYDp) in PTU-reduced goiter rats.

This study showed that PTU-induced marked decreases in body weight and rectal temperature with rough hair, slow response, and impaired liver function in goiter rats, with the characteristic of thyroid enlargement and hypothyroidism. Positive control Euthyrox treatment of 28 days altered the disordered thyroid hormone of hypothyroidism but showed no antigoitrous effects accompanied with impaired renal function. In contrast, both HYDf and HYDp treatment showed antigoitrous effects by inhibition of follicular cell hyperplasia and altering the disordered thyroid hormone of hypothyroidism. However, HYDf treatment displayed liver and renal injury, whereas HYDp treatment could protect the liver and renal function.

TSH and FT4 constitute most standard thyroid function tests and play a central role in the process of diagnosis and treatment of thyroid diseases such as hypothyroidism and thyrotoxicosis [17]. The thyroid gland plays a crucial role, through thyroid hormones (TH) synthesis, in the regulation of various physiological processes, including growth, development, and metabolic stability. The synthesis of TH is regulated by hypothalamus-pituitary-thyroid (HPT) axis in the negative feedback loop [18]. The HPT axis constitutes the main pathogenesis of goiter, with TSH being the strongest stimulatory factor for thyroid hyperplasia and proliferation in thyroid development, growth, and function. TSH can induce the expression and activate the critical genes involved in thyroid hormone formation sodium iodide symporter (NIS), Tg, and TPO [19–21]. The initial precursor of TH is Tg. TPO catalyzes the coupling of these iodotyrosine residues to form TH [22]. It was shown in the rat thyroid that Tg stored in the follicular lumen was a potent regulator of thyroid-specific gene expression to maintain the function of individual follicles [23]. Variants in the Tg gene or TPO gene have been linked to goiter, autoimmune thyroid diseases, and thyroid cancer [24–29].

In PTU-reduced goiter rats, TH levels are reduced and TRH levels are elevated. The TRH stimulates the pituitary to produce TSH, which in turn stimulates the thyroid gland to produce thyroid hormones [30] and induces the expression of TPO and Tg gene, and eventually leads to thyroid hyperplasia and proliferation. HYDf and HYDp could exhibit antigoitrous effects by regulating TH levels through alterations in HPT axis, reducing the evaluated TSH level. TSH plays a central role in promoting thyroid cell proliferation involved in the progress of thyroid growth [31]. The decreased TSH could inhibit the expression of TPO gene, one of the causes of thyroid dyshormonogenesis leading to hypothyroidism characterized by normal synthesis of TH and aberrant protein expression as reported [32, 33].

The decrease in relative organ weights is an indication of cell constriction [34] implying that PTU and HYDf could cause cellular constriction in both liver and kidney, whereas HYDp could not produce any effect on relative liver/kidney weights investigated.

The transaminases (AST and ALT) are well-known enzymes used as biomarkers to predict possible toxicity to the liver [35]. The elevation of AST as observed in the model control suggested possible hepatic injury. Administration of HYDp lowered the elevated activities of AST comparable to the model control and indicated the liver protection effect of HYDp. Renal function indices such as serum urea, creatinine, and uric acid can be employed to assess the toxic effects of chemicals on the kidney [36]. The elevations of the indices as observed in the model, Euthyrox, and HYDf treatment rats suggested possible renal injury; HYDp showed the nontoxic and kidney protection effect.

In summary, this study demonstrated that both HYDf and HYDp could exhibit antigoitrous effect through alterations in HPT axis and inhibition of the TPO gene expression; there was no difference in the antigoitrous effects between the two different species of* Sargassum* application in HYD. HYDp showed the nontoxic and liver/kidney protection effect, but HYDf could cause cellular constriction in both liver and kidney. The reason is unclear and further analysis is needed to clarify.

There are few published reports of experimental studies of antagonistic medicament herb pair HZ-GC; this study has evaluated the safety and efficacy of its application in HYD in goiter rats at the molecular, cellular, and whole level and compared the two species of* Sargassum* further. We provide a reliable way to clarify the possible mechanism of the antagonistic medicament herb pair HZ-GC for its application. The research on the potential mechanism is underway in our laboratory and will be reported shortly.

## Figures and Tables

**Table 1:** Compositions of HYD.

HYDf/HYDp	海藻玉*壶汤羊栖菜组*/海藻玉*壶汤*海蒿子组		
Latin name	Chinese name	Source	Daily adult dose (g)
*Glycyrrhiza uralensis *Fisch.	生*甘草*	Root and rhizome	24
*Sargassum fusiforme* (Harv.) Setch. /Sargassum pallidum (Turn.) C.Ag.	*羊栖菜* /海蒿子	Frond	24
*Pinellia ternata* (Thunb.) Breit.	*法半夏*	Tuber	9
*Fritillaria thunbergii *Miq.	*浙贝母*	Bulb	9
*Laminaria japonica* Aresch.	海带	Thallus	9
*Forsythia suspensa *(Thunb.) Vahl.	*连翘*	Fruit	9
*Ligusticum chuanxiong* Hort.	*川芎*	Rhizome	9
*Angelica pubescens *Maxim.* f. biserrata Shan et *Yuan	独活	Root	9
*Laminaria japonica *Aresch./Ecklonia kurome Okam.	昆布	Thallus	9
*Citrus reticulata* Blanco	*青皮*	Fruitlet	9
*Citrus reticulata* Blanco	*陈皮*	Pericarp	9
*Angelica sinensis* (Oliv.) Diels.	*当归*	Root	9

**Table 2:** Experimental schema.

Groups	Dose	Treatment
Normal	10 ml/kg/d	Rats received only normal feeds, distilled water, and gavage normal saline (1 ml/100 g body weight/day)
Model	0.01 g/kg/ml	Rats received PTU every two days, distilled water, and gavage distilled water (1 ml/100 g/day)
Euthyrox	0.01 g/kg/ml	Rats received Euthyrox and distilled water
HYDf	12.06 g/kg/d	Rats received HYDf (1 ml/100 g/day)
HYDp	12.06 g/kg/d	Rats received HYDp (1 ml/100 g/day)

**Table 3:** The primer sequences.

Primer	Sequence (5′ to 3′)	Length
TPO	FW: CTCCACGGATGCACTATCAG	180 bp
	RV: TTCTACCGA CGGAGGACAGA	
Tg	FW: GTGAACGCCTCTGTGACAGA	280 bp
	RV: ACGAAACCTGAGGACCGTCT	
GAPDH	FW: TTCACCACCATGGAGAAGGC	430 bp
	RV: ACTGTACGGCGGACCTCTTT	
